# A nomogram for predicting ARDS progression and need for invasive ventilation in COVID-19 patients using plasma hyaluronic acid and clinical scores

**DOI:** 10.3389/fcimb.2026.1762512

**Published:** 2026-04-16

**Authors:** Genhua Mu, Zhihao Nie, Yuan Xue, Shanshan Hou, Songping Xie, Chun Pan

**Affiliations:** 1 Department of Critical Care Medicine, Zhongda Hospital, School of Medicine, Southeast University, Nanjing, Jiangsu, China; 2 Department of Critical Care Medicine, Yancheng No.1 People’s Hospital, the Affiliated Hospital of the Medical School, Nanjing University, Yancheng, Jiangsu, China; 3 Department of Thoracic Surgery, Renmin Hospital of Wuhan University, Wuhan, Hubei, China; 4 Pharmacy Department, Yancheng No. 1 People’s Hospital, the Affiliated Hospital of the Medical School, Nanjing University, Yancheng, Jiangsu, China; 5 Department of Critical Care Medicine, Sichuan Academy of Medical Sciences and Sichuan People’s Hospital, Chengdu, Sichuan, China

**Keywords:** acute respiratory distress syndrome (ARDS), COVID-19, hyaluronic acid, invasive ventilation, nomogram

## Abstract

**Background:**

Acute respiratory distress syndrome (ARDS) is a major complication in hospitalized patients with coronavirus disease 2019 (COVID-19). Early identification of patients at increased risk of ARDS progression and invasive ventilation remains clinically important. This study aimed to investigate whether plasma hyaluronic acid (HA), alone or in combination with established clinical severity scores, could help identify ARDS, stratify severe disease, and assess the risk of endotracheal intubation in patients with COVID-19.

**Methods:**

This retrospective single-center cohort study included 502 adult patients with COVID-19 admitted between September 2022 and February 2023. Plasma HA levels were measured within 24 hours after admission. Demographic characteristics, comorbidities, laboratory findings, clinical severity scores, and outcome data were collected. Receiver operating characteristic analysis, multivariable logistic regression, and nomogram construction were used to evaluate the association of HA with ARDS diagnosis, severe ARDS stratification, and intubation risk.

**Results:**

This retrospective cohort analysis of 502 COVID-19 patients (361 in the non-ARDS group and 141 in the ARDS group) identified plasma hyaluronic acid (HA) as a potential biomarker for early ARDS diagnosis, severity stratification, and intubation risk assessment. HA levels were positively correlated with disease severity, with concentrations significantly increasing alongside ARDS severity, and showed good discrimination for ARDS (AUC = 0.904; sensitivity 81.6%, specificity 87.5% at a cut-off of 103 μg/L). HA also demonstrated excellent discrimination for severe ARDS, with an AUC of 0.953, comparable to that of the APACHE II and SOFA scores. It also showed good discrimination for endotracheal intubation risk in the overall cohort. The AUC for predicting intubation requirement reached 0.890 (sensitivity 76.6%, specificity 90.8%, NPV = 95.5% at a cut-off value of 140.1 μg/L). Integrating HA with clinical scores further improved model performance; incorporating age, lymphocyte count, CRP, CK, APACHE II, and SOFA into the baseline model increased the AUC for predicting severe ARDS from 0.960 to 0.973 (ΔAUC = 0.013). Furthermore, multivariable regression analysis showed that HA was independently associated with ARDS diagnosis (OR = 1.04, P = 0.007), severe ARDS (OR = 1.00, P < 0.001), and intubation risk (OR = 1.00, P = 0.011).

**Conclusion:**

Plasma HA was positively associated with ARDS severity and intubation risk in patients with COVID-19. HA may serve as a complementary biomarker for early risk stratification, particularly when used in combination with established clinical severity scores.

## 1 Introduction

Acute respiratory distress syndrome (ARDS) is characterized by acute hypoxemic respiratory failure, accompanied by diffuse pulmonary inflammation and bilateral edema, resulting from excessive alveolocapillary permeability in patients with non-cardiogenic pulmonary diseases (Ma et al., 2025). The incidence of ARDS is age dependent, increasing from 16/100,000 person-years for individuals 15–19 years of age to 306 per 100,000 person-years for individuals 75–84 years of age (Papazian et al., 2021). Despite advances in supportive care, ARDS remains a significant cause of morbidity and mortality in critically ill patients, with mortality rates of 35% for mild cases, 40% for moderate cases, and 45% for severe cases (Bellani et al., 2016). Pneumonia is the most common cause of ARDS, followed by extrapulmonary sepsis, aspiration, and trauma. Notably, SARS-CoV-2 infection is more likely to cause ARDS, which contributed to the COVID-19 pandemic (Attaway et al., 2021). Among those hospitalized for COVID-19, 15–30% typically develop COVID-19-associated acute respiratory distress syndrome (CARDS) (Aranda et al., 2021).

The endothelial glycocalyx (EG) is the innermost luminal layer of blood vessels, located on and within the vascular wall (Zhan et al., 2025). The glycocalyx is composed of proteoglycans, glycosaminoglycans, glycoproteins, and plasma proteins, with hyaluronic acid (HA) serving as a core constituent (Foote et al., 2022). Following endothelial injury, HA is shed from the glycocalyx into the systemic circulation, and its plasma concentration serves as a quantitative marker of endothelial damage severity (Prieto-Fernández et al., 2021). HA is an important component of the lung extracellular matrix that increases following infection with influenza or SARS-CoV-2 (Li et al., 2022; Dodd et al., 2024). Hellman et al. demonstrated that fragmented HA accumulates in the lungs of COVID-19 patients, with systemic HA levels being associated with reduced lung function 3–6 months after infection. This study provides novel insights into HA’ s role in COVID-19 pathology and its potential utility as a biomarker for disease severity (Hellman et al., 2024). Our previous research evaluated the prognostic value of plasma HA in assessing disease severity and outcomes in COVID-19 patients (Mu et al., 2025). Unlike previous studies focusing primarily on biomarker levels, the present study integrates plasma HA into nomogram-based models together with clinical severity scores to support individualized risk stratification in COVID-19-associated ARDS. The present study aimed to further investigate the association of plasma HA levels with ARDS identification, severe disease stratification, and endotracheal intubation risk in patients with CARDS. To our knowledge, this represents one of the larger cohorts evaluating HA in this clinical setting, and its findings may support clinical risk stratification and further evaluation of respiratory support strategies in CARDS patients.

## 2 Methods

### 2.1 Study design and setting

This retrospective cohort study enrolled 502 COVID-19 patients admitted to Yancheng No. 1 People’s Hospital, the Affiliated Hospital of the Medical School, Nanjing University, between September 2022 and February 2023. The study was approved by the hospital’s Ethics Committee (ethics number: 2023-K051). Trial registration number: ChiCTR2300071068 (website: https://www.chictr.org.cn/). All data were anonymized to protect patient confidentiality.

### 2.2 Study participants

This study included patients who met the following inclusion criteria: (1) diagnosed with COVID-19 and confirmed positive for the severe acute respiratory syndrome coronavirus 2 (SARS-CoV-2) nucleic acid based on throat swab reverse transcription polymerase chain reaction (RT-PCR) testing, in accordance with the Diagnosis and Treatment Protocol for Novel Coronavirus Pneumonia (Trial Version 9) issued by the National Health Commission; (2) nonpregnant adults; and (3) aged 18 to 80 years.

Patients with (1) non-infectious lung diseases, such as interstitial lung disease, pulmonary fibrosis, and chronic obstructive pulmonary disease (COPD); (2) chronic liver diseases, including hepatitis and liver cirrhosis; (3) chronic kidney disease (CKD) stages 4–5; (4) hematologic diseases; and (5) malignant tumors were excluded.

Based on the new global definition of ARDS (Matthay et al., 2024), patients were categorized into non-ARDS and ARDS groups. The ARDS group was further subdivided into the following subgroups: non-intubated (receiving only non-invasive ventilation, including high-flow nasal cannula oxygen therapy), mild ARDS, moderate ARDS, and severe ARDS.

ARDS patients were defined according to the global revised definition of ARDS (Matthay et al., 2024).Criteria that apply to specific ARDS categories: (1) Non-intubated ARDS: PaO_2_:FiO_2_ ≤300 mmHg or SpO_2_:FiO_2_ ≤315 (if SpO_2_ ≤97%) on HFNO with flow of ≥30 L/min or NIV/CPAP with at least 5 cmH_2_O end-expiratory pressure; (2) Intubated ARDS: Mild: 200 < PaO_2_:FiO_2_ ≤ 300 mmHg or 235 < SpO_2_:FiO_2_ ≤315 (if SpO_2_ ≤97%); Moderate: 100 < PaO_2_:FiO_2_ ≤200 mmHg or 148 < SpO_2_:FiO_2_ ≤235 (if SpO_2_ ≤97%); Severe: PaO_2_:FiO_2_ ≤100 mmHg or SpO_2_:FiO_2_ ≤148 (if SpO_2_ ≤97%).

### 2.3 Data collection

Plasma samples were collected within 24 hours of admission for all patients. Hyaluronic acid (HA) levels were measured using standard methods (ELK Biotechnology, ELK1234), alongside documentation of demographic characteristics, underlying conditions, laboratory parameters (leukocyte and platelet counts), disease severity scores (SOFA and APACHE II), and clinical outcomes during hospitalization (intubation requirement, duration of mechanical ventilation, and 28-day mortality, etc.). For the analysis of endotracheal intubation, patients were classified according to whether invasive mechanical ventilation was required during hospitalization. Data were extracted by two independent researchers from medical records and the hospital information system (HIS), with any scoring discrepancies resolved through arbitration by a senior physician.

### 2.4 Statistical methods

Statistical analyses were conducted using SPSS version 27.0 and R version 4.5.1, with data visualization performed using GraphPad Prism 10 and R 4.5.1. Continuous variables were presented as mean ± standard deviation and compared using independent samples t-tests when normally distributed, or as median (interquartile range) with comparisons made using the Mann-Whitney U test when normality assumptions were not met. Categorical variables were summarized as frequencies (percentages) and analyzed using chi-square or Fisher’s exact tests, as appropriate. Multiple group comparisons were conducted using the Kruskal-Wallis test with Bonferroni correction for *post hoc* analysis. Spearman correlation analysis was used to assess the relationship between hyaluronic acid (HA) levels and acute respiratory distress syndrome (ARDS) severity. Receiver operating characteristic (ROC) curve analysis evaluated the predictive performance of HA for ARDS, intubation status, and severe ARDS, reporting the area under the curve (AUC), sensitivity, specificity, and 95% confidence intervals. For two-sample comparisons of continuous variables, Welch’s t-test or the rank-sum test was applied as appropriate. Feature selection was performed using the Boruta algorithm combined with a random forest classifier; this method compared the importance scores of predictors against randomly generated shadow features to identify key predictors of ARDS and construct a predictive model. Model performance was assessed using ROC and calibration curves, with the AUC ranging from 0.5 (no discrimination) to 1.0 (perfect discrimination). Multivariate logistic regression analysis identified independent predictors and was used to develop a regression-based prediction model. Model discrimination and stability were evaluated via ROC curves and internal validation using bootstrap resampling (1,000 iterations). The corrected concordance index (c-index) was calculated from bootstrap samples to assess model performance. Calibration curves were plotted to evaluate the agreement between predicted probabilities and observed outcomes. Finally, decision curve analysis (DCA) was conducted to determine the net clinical benefit across different prediction thresholds.

## 3 Results

### 3.1 Population baseline data

**Figure 1:**
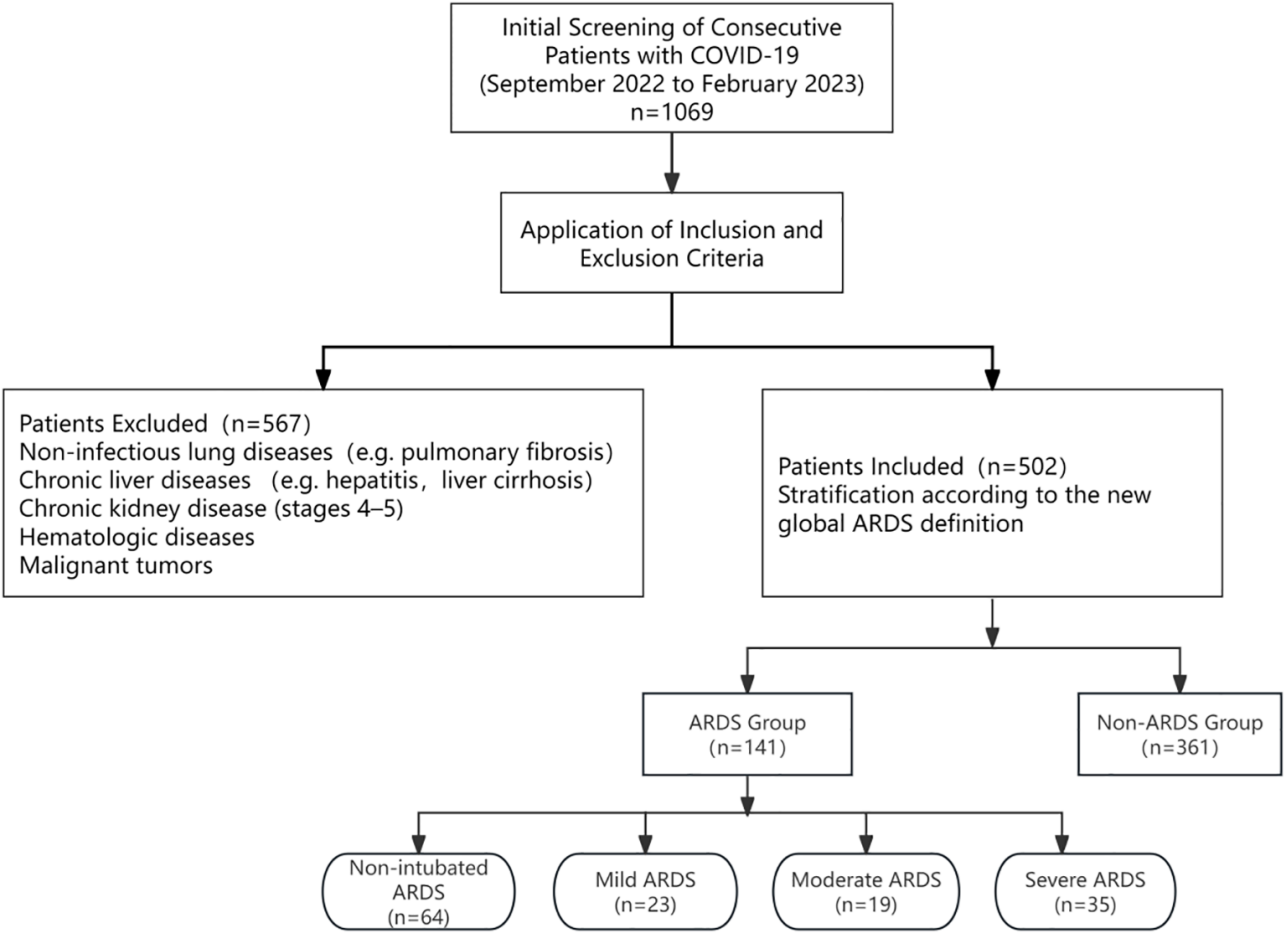
Flow chart of patients screening and cohort stratification.

This study included 502 patients categorized according to the new definition of ARDS into two groups: a non-ARDS group (N = 361) and an ARDS group (N= 141), further subdivided into non-intubated ARDS (N = 64), mild ARDS (N = 23), moderate ARDS (N = 19), and severe ARDS (N = 35) (**Figure 1**). Baseline analysis revealed significant differences in age (p < 0.001) and gender distribution (p < 0.001) across groups. The severe group exhibited the highest mean age (79 ± 11 years), while male patients predominated in the ARDS group (60.9%-80.0%). No statistically significant differences in BMI were observed across groups (p = 0.307). Laboratory findings: White blood cell counts increased with ARDS severity (p < 0.001), while lymphocyte counts decreased with increasing ARDS severity (p < 0.001). Inflammatory markers (C-reactive protein, D-dimer, HA) were significantly elevated in the ARDS group (all p < 0.001). Glucocorticoid use was near universal in the ARDS group (88.6%-100%) versus 74.8% in the non-ARDS group (p < 0.001). Disease severity scores (APACHE II and SOFA) progressively increased with ARDS severity (p < 0.001). Length of hospital stay exhibited complex patterns across groups (p < 0.001), with the most severe ARDS group having the longest duration of mechanical ventilation (p < 0.001) and ICU stay (p < 0.001) (**Table 1**). These findings highlight distinct demographic, clinical, and laboratory characteristics across different severity tiers of ARDS.

**Table 1:** Comparison of patient demographic and baseline characteristics stratified by ARDS severity.

Characteristic	Non-ARDS N = 361	ARDS group				p-value
		Non-intubated ARDS N = 64	Mild ARDS N = 23	Moderate ARDS N = 19	Severe ARDS N = 35	
Age, years (n%), Mean ± SD	67 ± 14	74 ± 12	77 ± 10	67 ± 21	79 ± 11	<0.001^1^
Gender (n%), n (%)						<0.001^2^
Male	184 (51.0%)	49 (76.6%)	14 (60.9%)	13 (68.4%)	28 (80.0%)	
Female	177 (49.0%)	15 (23.4%)	9 (39.1%)	6 (31.6%)	7 (20.0%)	
BMI, kg/m², Mean ± SD	24.5 ± 4.1	24.8 ± 4.3	24.2 ± 4.8	23.3 ± 3.2	23.3 ± 4.2	0.307^1^
Hypertension (n%), n (%)						0.175^2^
No	231 (64.0%)	31 (48.4%)	14 (60.9%)	11 (57.9%)	19 (54.3%)	
Yes	130 (36.0%)	33 (51.6%)	9 (39.1%)	8 (42.1%)	16 (45.7%)	
Diabetes mellitus (n%), n (%)						0.328^3^
No	285 (78.9%)	44 (68.8%)	17 (73.9%)	13 (68.4%)	26 (74.3%)	
Yes	76 (21.1%)	20 (31.3%)	6 (26.1%)	6 (31.6%)	9 (25.7%)	
COPD (n%), n (%)						0.197^3^
No	325 (90.0%)	59 (92.2%)	17 (73.9%)	17 (89.5%)	32 (91.4%)	
Yes	36 (10.0%)	5 (7.8%)	6 (26.1%)	2 (10.5%)	3 (8.6%)	
Glucocorticoid Used (n%), n (%)						<0.001^3^
Yes	270 (74.8%)	64 (100.0%)	21 (91.3%)	17 (89.5%)	31 (88.6%)	
No	91 (25.2%)	0 (0.0%)	2 (8.7%)	2 (10.5%)	4 (11.4%)	
White Blood Cell Count, 10^9^/L, Mean ± SD	6.7 ± 3.2	9.2 ± 4.9	9.3 ± 6.1	9.5 ± 5.1	10.6 ± 5.2	<0.001^1^
Platelet Count, 10^9^/L, Mean ± SD	208 ± 89	195 ± 81	205 ± 83	178 ± 92	175 ± 85	0.150^1^
Red Blood Cell Count, 10¹²/L, Mean ± SD	4.19 ± 0.58	4.26 ± 0.71	4.32 ± 0.78	4.11 ± 0.52	4.12 ± 0.75	0.664^1^
Lymphocyte, 10^9^/L, Mean ± SD	1.17 ± 0.96	0.82 ± 0.46	0.67 ± 0.40	0.59 ± 0.27	0.77 ± 0.90	<0.001^1^
C-reactive protein, mg/L, Median (Q1, Q3)	39 (16, 62)	80 (42, 102)	93 (43, 115)	115 (54, 146)	86 (36, 172)	<0.001^4^
D-dimer, mg/L, Median (Q1, Q3)	0.51 (0.31, 0.96)	1.00 (0.50, 3.48)	1.17 (0.56, 2.43)	2.00 (0.81, 4.94)	2.10 (0.80, 4.78)	<0.001^4^
Creatine Kinase, U/L, Median (Q1, Q3)	78 (51, 121)	78 (37, 115)	101 (59, 168)	122 (56, 326)	175 (87, 533)	<0.001^4^
ALT, U/L, Median (Q1, Q3)	25 (18, 37)	28 (19, 42)	24 (16, 35)	29 (21, 35)	34 (23, 52)	0.033^4^
Total Bilirubin, μmol/L, Median (Q1, Q3)	11.2 (8.5, 15.2)	13.5 (10.6, 18.2)	16.6 (14.2, 20.0)	15.5 (9.4, 19.8)	14.3 (9.4, 17.3)	<0.001^4^
Hyaluronic Acid, μg/L, Median (Q1, Q3)	72 (61, 90)	149 (104, 269)	113 (85, 259)	271 (140, 840)	405 (231, 1,726)	<0.001^4^
Apache II scores, Median (Q1, Q3)	0.0 (0.0, 0.0)	14.5 (13.0, 16.0)	16.0 (14.0, 17.0)	19.0 (16.0, 20.0)	19.0 (17.0, 19.0)	<0.001^4^
SOFA scores, Median (Q1, Q3)	0.00 (0.00, 0.00)	6.00 (5.00, 7.00)	6.00 (4.00, 7.00)	8.00 (7.00, 9.00)	8.00 (7.00, 9.00)	<0.001^4^
Endotracheal Intubation (n%), n (%)						<0.001^3^
No	361 (100.0%)	64 (100.0%)	0 (0.0%)	0 (0.0%)	0 (0.0%)	
Yes	0 (0.0%)	0 (0.0%)	23 (100.0%)	19 (100.0%)	35 (100.0%)	
MV Mechanical Ventilation, days, Median (Q1, Q3)	0.00 (0.00, 0.00)	1.00 (1.00, 1.00)	0.00 (0.00, 0.00)	0.00 (0.00, 6.00)	3.00 (0.00, 6.00)	<0.001^4^
The duration of ICU, days, Median (Q1, Q3)	0.00 (0.00, 0.00)	0.00 (0.00, 0.50)	0.00 (0.00, 0.00)	2.00 (0.00, 8.00)	3.00 (0.00, 8.00)	<0.001^4^
LOS Length of stay, days, Median (Q1, Q3)	9 (7, 12)	0 (0, 1)	9 (6, 11)	13 (8, 20)	9 (5, 17)	<0.001^4^

^1^One-way analysis of means.

^2^Pearson's Chi-squared test.

^3^Fisher's exact test.

^4^Kruskal-Wallis rank sum test.

ARDS, acute respiratory distress syndrome; BMI, body mass index; WBC, white blood cell count; PLT, platelet count; RBC, red blood cell count; CRP, C-reactive protein; CK, creatine kinase; ALT, alanine aminotransferase; TB, total bilirubin; HA, hyaluronic acid; APACHE II, Acute Physiology and Chronic Health Evaluation II; SOFA, Sequential Organ Failure Assessment; EI, endotracheal intubation; MV, mechanical ventilation; ICU, intensive care unit; LOS, length of stay; SD, standard deviation; Q1, first quartile; Q3, third quartile.

### 3.2 Analysis of the correlation and diagnostic value of hyaluronic acid levels with ARDS severity

**Figure 2:**
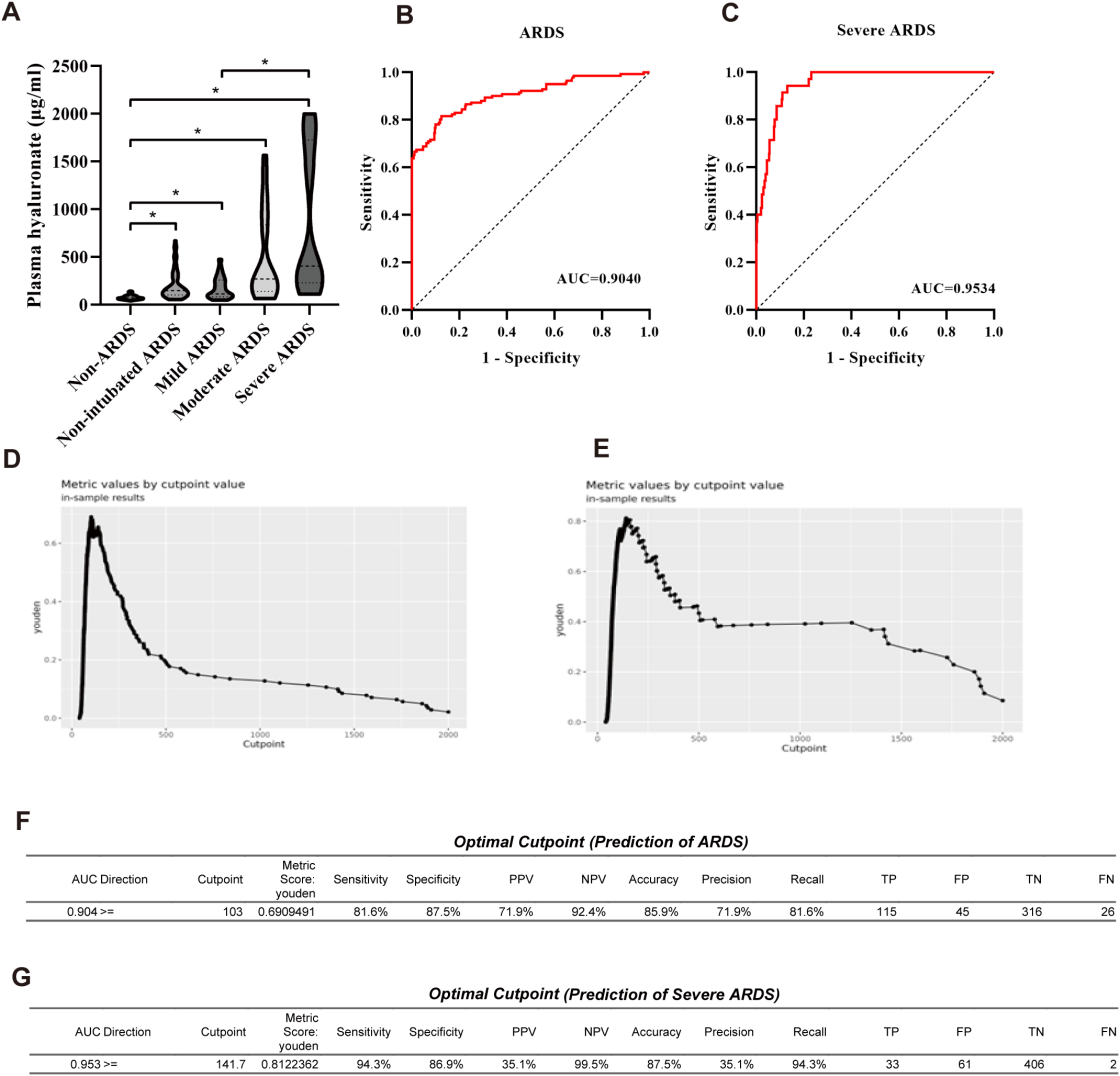
Diagnostic and stratification value of HA for ARDS. **(A)** Kruskal-Wallis test analysis between subgroups with different plasma HA levels; **(B)** ROC curve for HA diagnosis of ARDS; **(C)** ROC curve for HA prediction of severe ARDS; **(D)** Optimal cut-off value for HA in diagnosing ARDS; **(E)** Optimal cut-off value for HA in diagnosing severe ARDS; **(F)** Table of optimal cut-off values for HA in diagnosing ARDS; **(G)** Table of optimal cut-off values for HA in diagnosing severe ARDS.

To investigate the correlation between plasma HA levels and ARDS severity, we performed Spearman’s correlation analysis on the entire patient cohort without subgrouping. The results demonstrated a strong positive correlation between HA levels and ARDS severity (*r* = 0.641, *P* < 0.001), indicating a consistent increase in HA levels with worsening ARDS severity. Subsequently, we compared differences between subgroups (**Figure 2A**). The Kruskal–Wallis test revealed significant overall differences in HA levels across the non-ARDS, non-intubated ARDS, mild, moderate, and severe ARDS groups (P < 0.001). Bonferroni-corrected pairwise comparisons showed significantly lower HA levels in the non-ARDS group compared to all ARDS subgroups (P < 0.001 for all comparisons). Furthermore, HA levels in the mild ARDS and non-intubated subgroups were significantly lower than those in the severe ARDS subgroup (P = 0.034 and 0.031, respectively). However, no statistically significant differences in HA levels were observed between adjacent ARDS subgroups (e.g., mild vs. non-intubated, mild vs. moderate; P > 0.05). These findings suggest that HA may serve as an early biomarker for ARDS development and may also have potential value for severe ARDS stratification.

Furthermore, we evaluated the diagnostic efficacy of hyaluronic acid (HA) for ARDS (**Figure 2B**). ROC curve analysis revealed that HA’s area under the curve (AUC) was 0.904 (standard error = 0.017, P < 0.001), with a 95% CI of 0.870**–**0.938, indicating excellent diagnostic performance for ARDS. The optimal cut-off value for HA in diagnosing ARDS (**Figures 2D, F**) was 103 μg/L. At this threshold, HA demonstrated a sensitivity of 81.6% and a specificity of 87.5%, with a false negative rate of 18.4% (1–sensitivity) and a false positive rate of 12.5% (1–specificity). ROC analysis of HA’s diagnostic performance for severe ARDS patients (**Figure 2C**) demonstrated excellent discrimination, with an AUC of 0.953 (95% CI: 0.931–0.976). The corresponding threshold distribution is shown in **Figures 2E, G**. These findings suggest that HA may serve as a biomarker for ARDS diagnosis supporting early identification and severity stratification.

### 3.3 Association of HA with endotracheal intubation risk in the overall cohort

**Figure 3:**
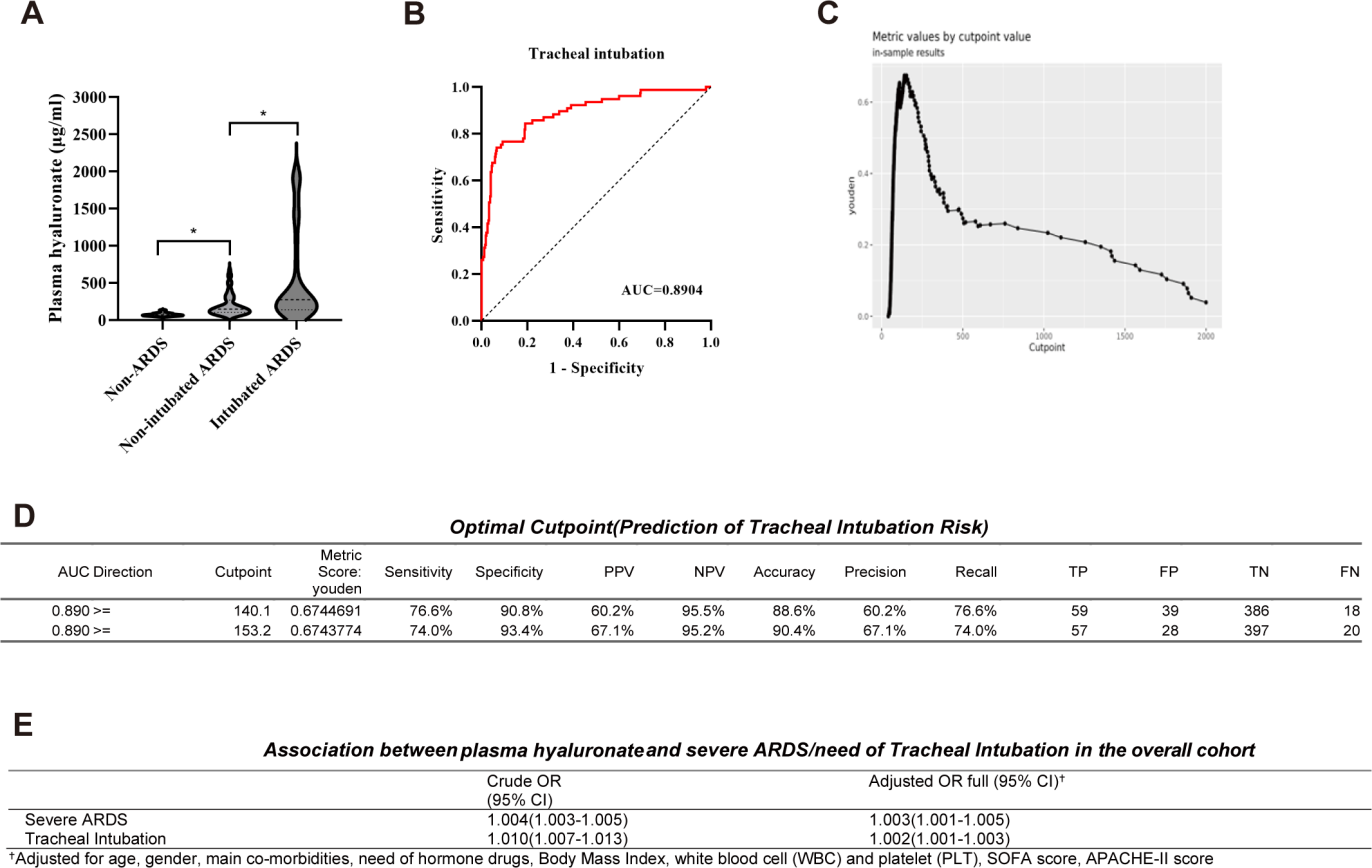
Association of HA with endotracheal intubation risk in the overall cohort. **(A)** Mann-Whitney U test comparing HA levels between intubated and non-intubated groups; **(B)** ROC curve for HA predicting endotracheal intubation; **(C)** Optimal cut-off value of HA for predicting endotracheal intubation; **(D)** Table of optimal cut-off values of HA for predicting endotracheal intubation; **(E)** Unadjusted and adjusted odds ratios for the associations of plasma HA with severe ARDS and tracheal intubation in the overall cohort.

To investigate the association of hyaluronic acid (HA) with endotracheal intubation, patients were categorized into intubated and non-intubated groups according to whether invasive mechanical ventilation was required during hospitalization. The Mann-Whitney U test for independent samples was used to compare HA levels between the groups. Results demonstrated a significant difference in HA distribution between the groups (P < 0.001) (**Figure 3A**).

To evaluate HA’s discriminative performance for tracheal intubation, ROC curve analysis was performed (**Figure 3B**). The area under the curve (AUC) for at risk of tracheal intubation in the overall cohort was 0.890 (95% CI: 0.846–0.935, P < 0.001). The optimal cut-off value was determined by maximizing the Youden index (**Figures 3C, D**). At a cut-off of 140.1, the model demonstrated a sensitivity of 76.6% (miss rate 23.4%), specificity of 90.8% (false positive rate 9.2%), and a negative predictive value (NPV) of 95.5%. Increasing the cut-off to 153.2 reduced sensitivity to 74.0% but increased specificity to 93.4%, with the positive predictive value (PPV) rising from 60.2% to 67.1%. The ROC model showed good discriminative ability (AUC = 0.890) and a high NPV (>95%), indicating that HA may be useful for identifying low-risk patients who are unlikely to require intubation. However, a certain risk of misclassification remains in positive predictions, as reflected by the PPV range of 60.2% to 67.1%.

Subsequent multivariable regression analysis, adjusted for confounding factors—including age, gender, comorbidities, corticosteroid use, BMI, WBC, PLT, SOFA score, and APACHE-II score—revealed the following (**Figure 3E**): For each 1 μg/L increase in plasma HA levels, the adjusted odds ratio (OR) for severe ARDS was 1.003 (95% CI: 1.001–1.005), and the adjusted OR for intubation risk was 1.002 (95% CI: 1.001–1.003). These findings indicate a modest yet independent positive association between elevated plasma HA levels and the risk of severe ARDS and the need for mechanical ventilation.

### 3.4 Comparison of HA and clinical scoring models for ARDS diagnosis, severity stratification, and intubation prediction

**Figure 4:**
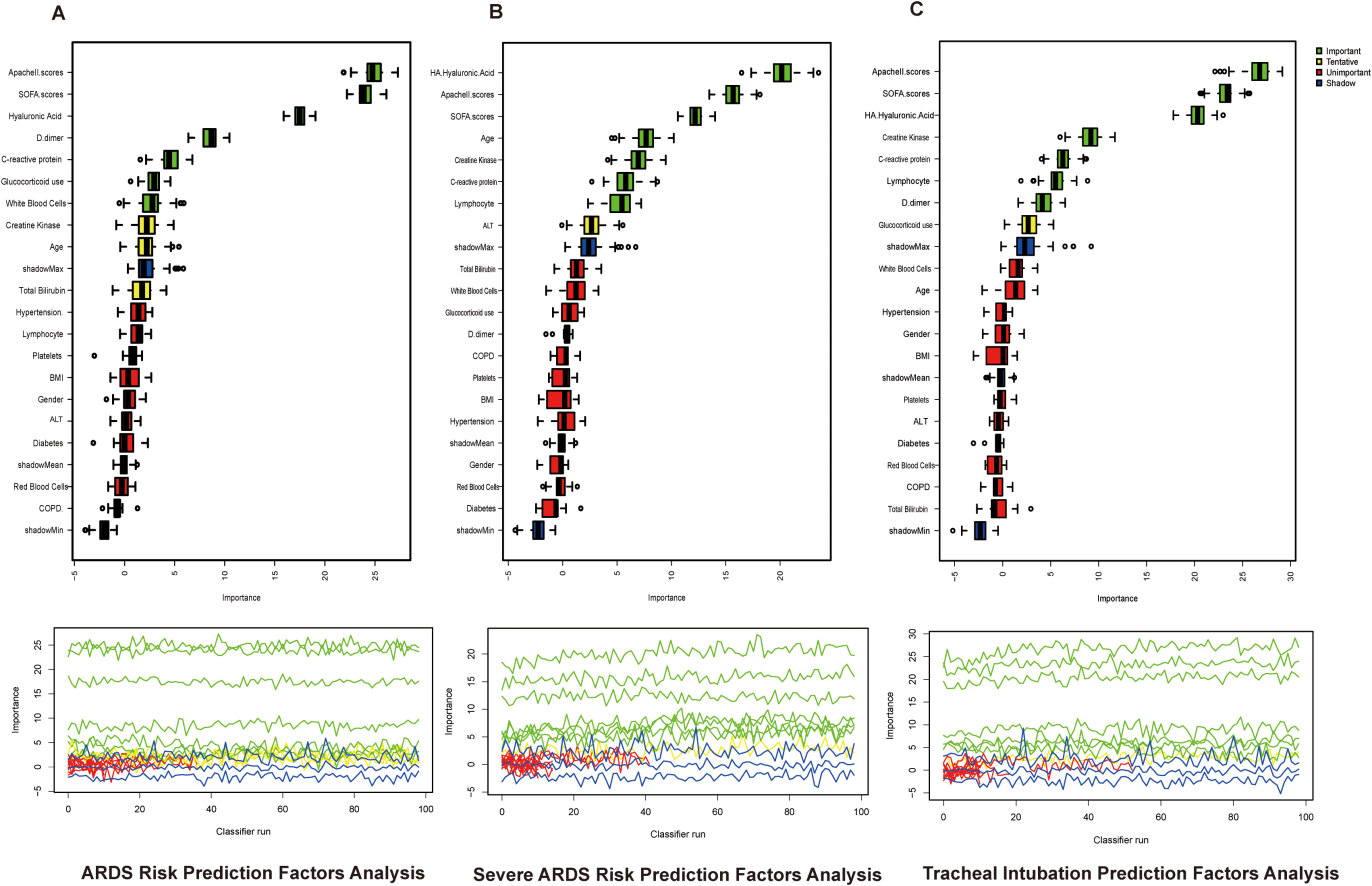
Key predictors for ARDS diagnosis, ARDS severity prediction, and tracheal intubation in the overall cohort. **(A)** Key predictors in the ARDS occurrence risk prediction model; **(B)** Key predictors in the severe ARDS occurrence risk prediction model; **(C)** Key predictors in the tracheal intubation risk prediction model.

To identify key predictors for diagnosing ARDS, stratifying its severity, and anticipating the need for tracheal intubation, we employed the Boruta feature selection algorithm based on a random forest framework to screen commonly used clinical indicators (e.g., inflammatory cytokines, biochemical markers, underlying conditions). In the ARDS risk prediction model (**Figure 4A**), the APACHE II score, SOFA score, hyaluronic acid (HA) levels, D-dimer, C-reactive protein, corticosteroid use, and white blood cell count were identified as key features. For the severe ARDS risk prediction model (**Figure 4B**), key features included hemoglobin concentration, APACHE II score, SOFA score, age, creatine kinase, C-reactive protein, and lymphocyte count. In the tracheal intubation risk prediction model (**Figure 4C**), the key features were APACHE II score, SOFA score, HA level, creatine kinase, C-reactive protein, lymphocyte count, and D-dimer. These Boruta-selected variables were considered candidate predictors, and the final multivariable models were established by combining feature selection results with clinical relevance.

**Figure 5:**
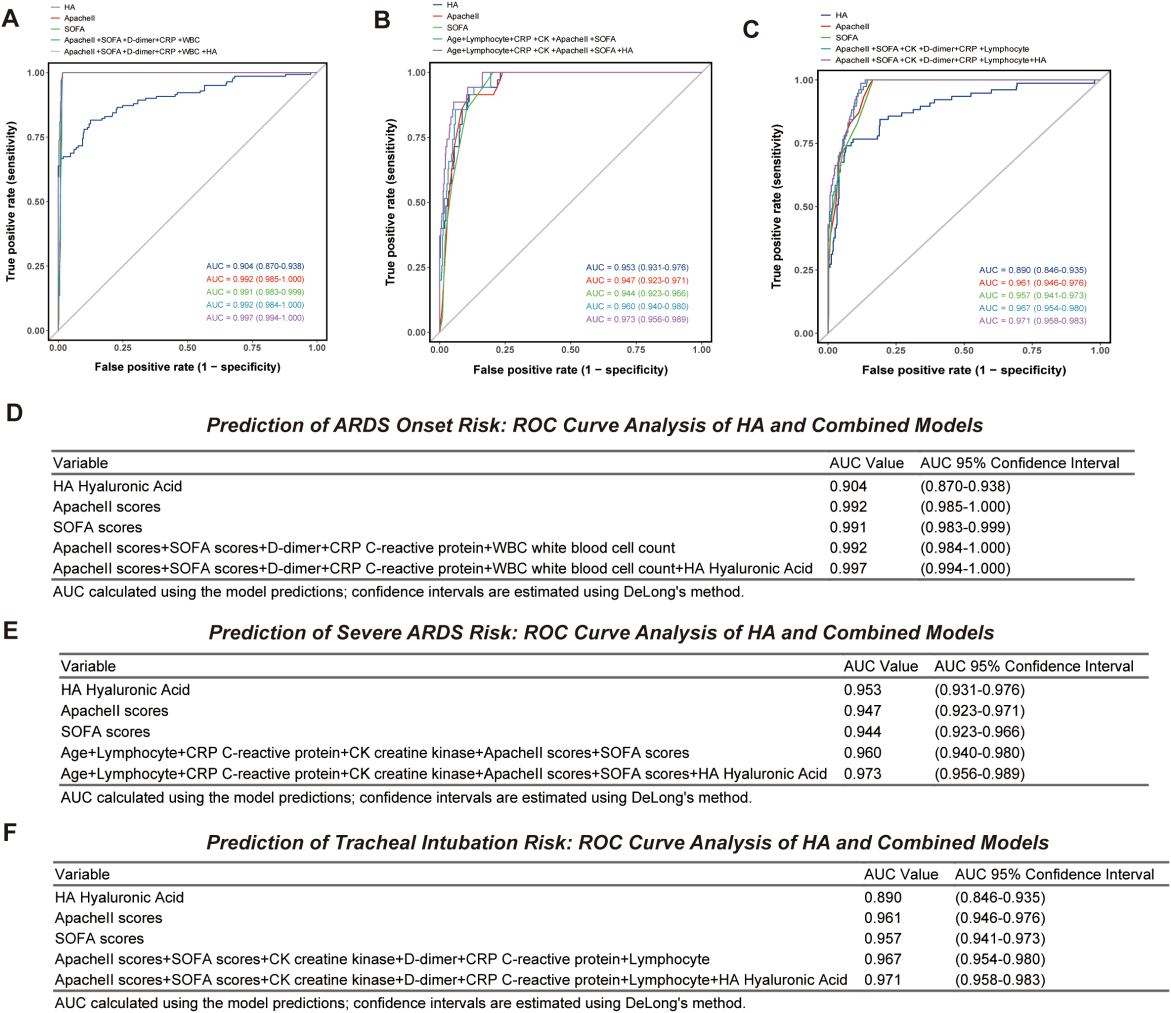
Independent predictive value of HA and its synergistic effects with clinical indicators. **(A)** Changes in ARDS diagnostic performance and AUC table for HA combined with multifactorial models; **(B)** Changes in performance and AUC table for severe ARDS stratification using HA combined with multifactorial models; **(C)** Changes in performance and AUC table for predicting endotracheal intubation using HA combined with multifactorial models; **(D)** Table of AUC values for HA and combined multifactorial models in predicting ARDS; **(E)** Table of AUC values for HA and combined multifactorial models in predicting severe ARDS; **(F)** Table of AUC values for HA and combined multifactorial models in predicting endotracheal intubation.

To further validate HA’s independent discriminative value and its synergistic effects with clinical indicators, we compared the efficacy of HA as a single marker versus HA combined with classical scoring models in predicting ARDS-related outcomes (**Figures 5A–C**). ROC curve analysis revealed that HA’s AUC for diagnosing ARDS was 0.904 (95% CI: 0.870–0.938), and that it also showed excellent discrimination for severe ARDS (AUC = 0.953, 95% CI: 0.931–0.976). The AUC for predicting endotracheal intubation was 0.890 (95% CI: 0.846–0.935). By comparison, classic scoring systems demonstrated excellent discrimination in diagnosing ARDS (AUC 0.992 for APACHE II and 0.991 for SOFA) and predicting tracheal intubation (AUC 0.961 for APACHE II and 0.957 for SOFA, both > 0.95). However, their AUCs for predicting severe ARDS were slightly lower—0.947 for APACHE II and 0.944 for SOFA—both marginally below that of HA alone. Further integration of HA into multivariate combination models modestly improved predictive performance across all outcomes. In the ARDS diagnosis model, the baseline combination (APACHE II + SOFA + D-dimer + CRP + WBC) achieved an AUC of 0.992, which increased to 0.997 (ΔAUC = +0.005) upon HA inclusion. In the severe ARDS prediction model, the baseline combination (age + lymphocyte count + CRP + creatine kinase + APACHE II + SOFA) yielded an AUC of 0.960, rising to 0.973 after integrating HA (ΔAUC = +0.013). In the tracheal intubation prediction model, the baseline combination (APACHE II + SOFA + creatine kinase + D-dimer + CRP + lymphocyte count) had an AUC of 0.967, which increased to 0.971 after incorporating HA (ΔAUC = +0.004). In summary, HA showed the numerically highest AUC among the single predictors for severe ARDS (AUC = 0.953). Its inclusion in multivariate models was associated with modest improvements in model performance across all outcomes.

### 3.5 Construction and performance evaluation of nomogram models for ARDS diagnosis, severity grading, and intubation risk prediction based on HA and clinical scores

**Figure 6:**
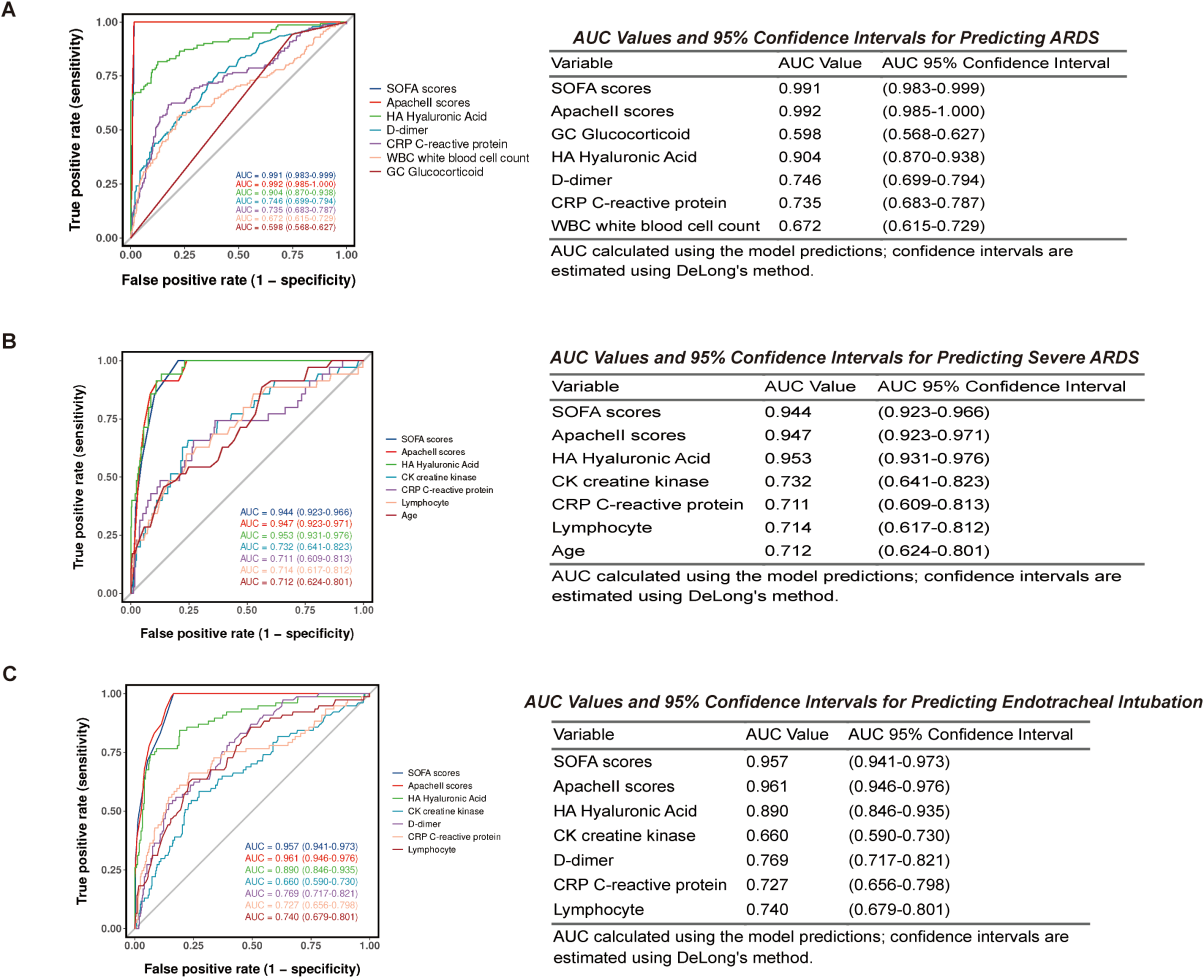
Diagnostic performance of HA and clinical scores for ARDS diagnosis, severity, and risk of endotracheal intubation. **(A)** ROC curves and AUC values for each independent factor in diagnosing ARDS; **(B)** ROC curves and AUC values for each independent factor in stratifying severe ARDS; **(C)** ROC curves and AUC values for each independent factor in predicting endotracheal intubation in the overall cohort.

To further explore the potential clinical utility of these predictors, we developed nomogram models. The initial assessment of diagnostic efficacy for ARDS is shown in **Figure 6A**. The SOFA score and APACHE II score demonstrated the highest diagnostic predictive efficacy for ARDS, with AUC values of 0.991 (95% CI: 0.983–0.999) and 0.992 (95% CI: 0.985–1.000), respectively. HA showed the next highest predictive efficacy with an AUC of 0.904 (95% CI: 0.870–0.938), indicating good diagnostic value. D-dimer and CRP exhibited moderate predictive capability, with AUC values of 0.746 (95% CI: 0.699–0.794) and 0.735 (95% CI: 0.683–0.787), respectively. In contrast, the predictive efficacy of WBC and glucocorticoid use (GC) was relatively low, with AUC values of 0.672 (95% CI: 0.615–0.729) and 0.598 (95% CI: 0.568–0.627) respectively.

Subsequently, we further evaluated the diagnostic efficacy of each factor for severe ARDS (**Figure 6B**). ROC curve analysis revealed significant differences in the discriminatory power of the predictive variables for the severe ARDS group. Among these, HA demonstrated the numerically highest discrimination, with an AUC of 0.953 (95% CI: 0.931–0.976). The APACHE II score (AUC = 0.947, 95% CI: 0.923–0.971) and SOFA score (AUC = 0.944, 95% CI: 0.923–0.966) also exhibited excellent discriminatory capability (AUC > 0.9). In contrast, creatine kinase (AUC = 0.732), C-reactive protein (AUC = 0.711), lymphocyte count (AUC = 0.714), and age (AUC = 0.712) showed comparatively lower predictive efficacy. All these markers had AUC values below 0.8 with wider 95% confidence intervals, indicating limited discriminatory capacity for severe ARDS.

Finally, we assessed the predictive value of each factor for endotracheal intubation in the overall cohort (**Figure 6C**). ROC curve analysis revealed that the SOFA score and APACHE II score demonstrated the highest predictive efficacy for endotracheal intubation (EI), with AUC values of 0.957 (95% CI: 0.941–0.973) and 0.961 (95% CI: 0.946–0.976), respectively. Hyaluronic acid (HA) showed the next highest predictive capability, with an AUC of 0.890 (95% CI: 0.846–0.935), while D-dimer, lymphocyte count, and C-reactive protein exhibited moderate predictive efficacy, with AUC values of 0.769 (95% CI: 0.717–0.821), 0.740 (95% CI: 0.679–0.801), and 0.727 (95% CI: 0.656–0.798), respectively. Creatine kinase demonstrated relatively weaker predictive capability, with an AUC of 0.660 (95% CI: 0.590–0.730). These findings indicate that both the SOFA score and APACHE II score possess high diagnostic value in predicting the need for endotracheal intubation.

Subsequently, we conducted a multivariate logistic regression analysis to examine the relationship between various factors and the final outcome. The results indicated that, regarding ARDS diagnosis (**Table 2**), the APACHE II score (OR = 2.14, 95% CI: 1.44–3.18, P < 0.001) and hyaluronic acid levels (OR = 1.04, 95% CI: 1.01–1.07, P = 0.007) were significantly associated with an increased risk of ARDS. Although the glucocorticoid-treated group (OR = 3.55, 95% CI: 0.04–333.09, P = 0.584) showed a trend toward higher risk, this did not reach statistical significance. The SOFA score (OR = 0.55, 95% CI: 0.23–1.30, P = 0.172), D-dimer (OR = 0.99, 95% CI: 0.96–1.03, P = 0.689), C-reactive protein (OR = 1.01, 95% CI: 0.99–1.03, P = 0.289), and white blood cell count (OR = 0.98, 95% CI: 0.83–1.16, P = 0.837) were not significantly associated with ARDS risk.

**Table 2:** Results of multivariate logistic regression (prediction of ARDS).

Characteristic	N	Event N	Odds ratio	95% confidence interval	P-value
SOFA scores	502	141	0.55	0.23, 1.30	0.172
Apache II scores	502	141	2.14	1.44, 3.18	<0.001
Glucocorticoid used					
No	99	8	—	—	
Yes	403	133	3.55	0.04, 333.09	0.584
HA	502	141	1.04	1.01, 1.07	0.007
D-dimer	502	141	0.99	0.96, 1.03	0.689
C-reactive protein	502	141	1.01	0.99, 1.03	0.289
White Blood Cell count	502	141	0.98	0.83, 1.16	0.837

CI, Confidence Interval; OR, Odds Ratio.

Regarding the diagnosis of severe ARDS (**Table 3**), HA levels were significantly associated with the occurrence of severe ARDS (OR = 1.00, 95% CI: 1.00–1.00, P < 0.001), while creatine kinase levels also demonstrated statistical significance (OR = 1.00, 95% CI: 1.00–1.00, P = 0.029). Age showed a marginally significant association with severe ARDS (OR = 1.05, 95% CI: 1.00–1.11, P = 0.050). SOFA score (OR = 1.54, 95% CI: 0.89–2.67), APACHE II score (OR = 1.19, 95% CI: 0.89–1.59), lymphocyte count (OR = 1.45, 95% CI: 0.83–2.54), and C-reactive protein (CRP) level (OR = 1.00, 95% CI: 0.99–1.00) showed no significant association with the occurrence of severe ARDS (all P-values > 0.05).

**Table 3:** Results of multivariate logistic regression (prediction of severe ARDS).

Characteristic	N	Event N	Odds ratio	95% confidence interval	P-value
SOFA scores	502	35	1.54	0.89, 2.67	0.123
Apache II scores	502	35	1.19	0.89, 1.59	0.236
HA	502	35	1.00	1.00, 1.00	<0.001
Creatine Kinase	502	35	1.00	1.00, 1.00	0.029
C-reactive protein	502	35	1.00	0.99, 1.00	0.337
Lymphocyte	502	35	1.45	0.83, 2.54	0.189
Age	502	35	1.05	1.00, 1.11	0.050

CI, Confidence Interval; OR, Odds Ratio.

In predicting the need for intubation in the overall cohort (**Table 4**), the APACHE II score (OR = 1.35, 95% CI: 1.10–1.66, P = 0.004), hyaluronic acid (OR = 1.00, 95% CI: 1.00–1.00, p = 0.011), and creatine kinase (CK) (OR = 1.00, 95% CI: 1.00–1.00, P = 0.042) were significantly associated with the risk of tracheal intubation. In contrast, the SOFA score (OR = 1.22, 95% CI: 0.81–1.84, P = 0.351), D-dimer (OR = 0.96, 95% CI: 0.92–1.01, P = 0.094), C-reactive protein (OR = 1.00, 95% CI: 1.00–1.01, P = 0.234), and lymphocyte count (OR = 0.65, 95% CI: 0.32–1.31, P = 0.225) were not statistically significant. In summary, HA was independently associated with ARDS diagnosis, severe ARDS stratification, and endotracheal intubation risk.

**Table 4:** Results of multivariate logistic regression (prediction of tracheal intubation risk).

Characteristic	N	Event N	Odds ratio	95% confidence interval	P-value
SOFA scores	502	77	1.22	0.81, 1.84	0.351
Apache II scores	502	77	1.35	1.10, 1.66	0.004
HA	502	77	1.00	1.00, 1.00	0.011
Creatine Kinase	502	77	1.00	1.00, 1.00	0.042
D-dimer	502	77	0.96	0.92, 1.01	0.094
C-Reactive Protein	502	77	1.00	1.00, 1.01	0.234
Lymphocyte	502	77	0.65	0.32, 1.31	0.225

CI, Confidence Interval; OR, Odds Ratio.

**Figure 7:**
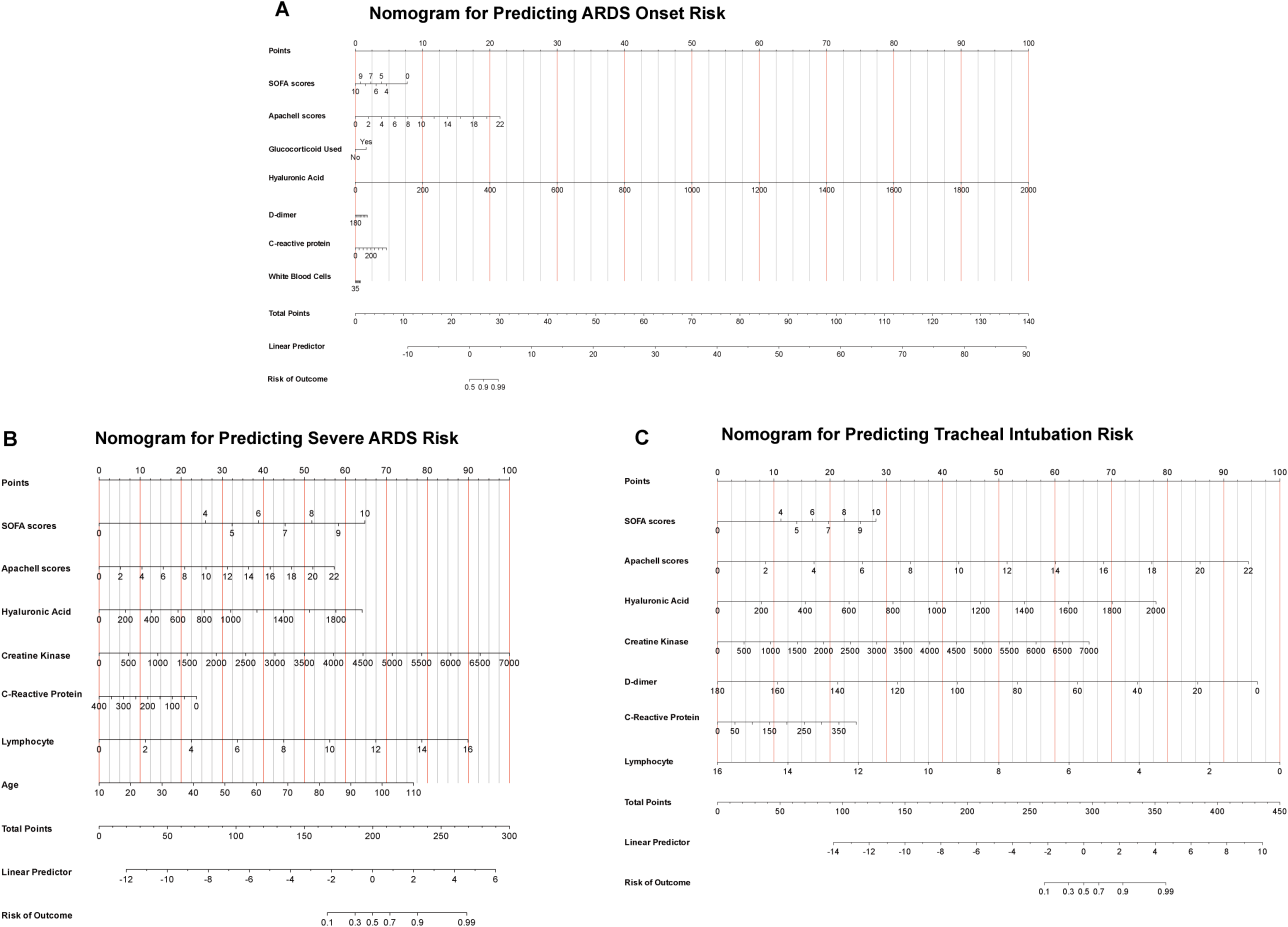
Nomograms for individualized prediction of ARDS diagnosis, severe ARDS, and endotracheal intubation risk. **(A)** ARDS onset risk prediction nomogram: Each variable is assigned a score based on its contribution to ARDS risk, with higher scores indicating greater predictive weight. Total scores range from 0 to 140 points; cumulative totals estimate linear predicted values and final ARDS onset risk. **(B)** Severe ARDS risk prediction nomogram. Variable scores accumulate to form a total score (0–300 points), yielding a linear predicted value and the probability of severe ARDS occurrence. **(C)** Endotracheal intubation risk prediction nomogram in the overall cohort. Total scores range from 0 to 450 points, derived from the sum of variable scores; higher scores indicate greater intubation risk.

Based on the results described above, we developed three nomograms to quantify ARDS diagnosis (**Figure 7A**), stratify severe ARDS (**Figure 7B**), and assess endotracheal intubation risk in the overall cohort (**Figure 7C**).

In the ARDS diagnosis nomogram (**Figure 7A**), the SOFA score, APACHE II score, plasma HA, D-dimer, CRP, WBC, and glucocorticoid use were each assigned a score reflecting their contribution to ARDS risk, with higher scores indicating greater predictive weight. The total score ranges from 0 to 140 points. By summing these scores, a linear prediction value and the corresponding risk of ARDS onset can be estimated, providing a visualization tool for individualized risk stratification.

The severe ARDS risk prediction nomogram (**Figure 7B**) incorporates the SOFA score, APACHE II score, HA, creatine kinase (CK), CRP, lymphocyte count, and age. Similar to the ARDS onset model, individual variable scores are summed to generate a total score ranging from 0 to 300 points, which is then mapped to linear predicted values and the probability of severe ARDS occurrence.

**Figure 8:**
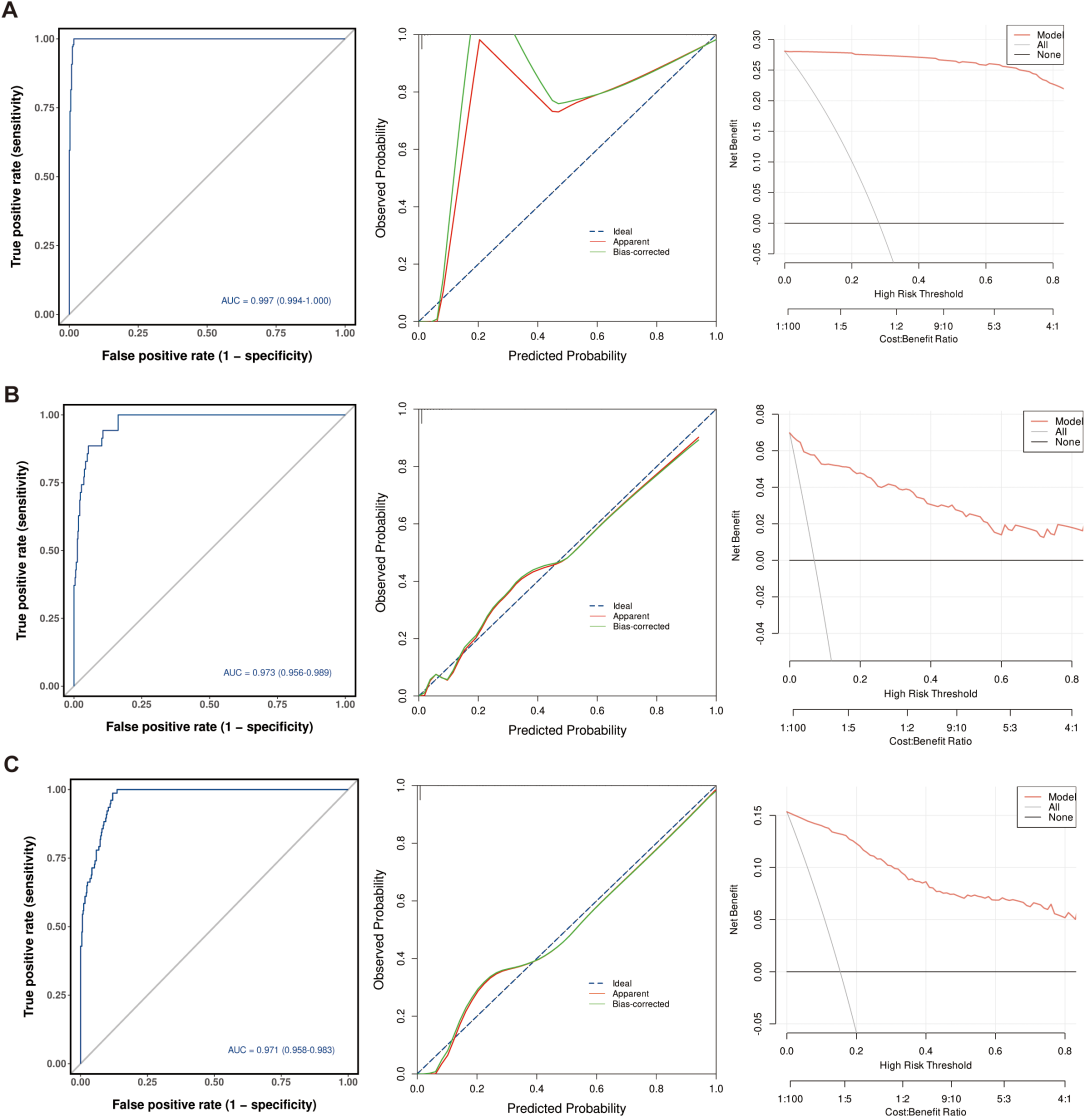
Nomogram efficacy evaluation. **(A)** ARDS onset risk prediction nomogram-ROC curve, calibration curve, and decision curve analysis (DCA); **(B)** Severe ARDS risk prediction nomogram-ROC curve, calibration curve, and decision curve analysis; **(C)** Nomogram for predicting the risk of tracheal intubation in the overall cohort: ROC curve, calibration curve, and decision curve analysis.

The endotracheal intubation risk nomogram for the overall cohort (**Figure 7C**) incorporates the SOFA score, APACHE II score, HA, CK, D-dimer, CRP, and lymphocyte count. The total score ranges from 0 to 450 points, calculated by summing the individual variable scores, with higher scores indicating an increased risk of intubation. The model converts the total score into a linear predicted value and then into the final probability of intubation, thereby providing a visual tool for individualized risk assessment. Finally, we evaluated the predictive performance of the three constructed nomograms—for ARDS diagnosis, severe ARDS stratification, and intubation risk—using ROC curves, calibration curves, and decision curve analysis (DCA). The results are as follows:

1. Validation of the ARDS onset risk nomogram (**Figure 8A**). Discrimination (ROC curve): The area under the ROC curve (AUC) for this nomogram was 0.997 (95% CI: 0.994–1.000). Calibration (calibration curve): The fitted curve of observed probability versus predicted probability (green solid line) closely approximates the ideal reference line (blue dashed line), indicating strong agreement between the model’s predicted probabilities and the actual risk of onset. Clinical utility (decision curve): When the threshold probability (high-risk threshold) ranges from 0.02 to 0.6, the net benefit of using this nomogram (red curve) is significantly greater than that of the “All” (full intervention) or “None” (no intervention) strategies, suggesting potential clinical utility.

2. Validation of the Severe ARDS Risk Nomogram (**Figure 8B**). Discrimination (ROC curve): The model achieved an AUC of 0.973 (95% CI: 0.956–0.989), demonstrating excellent discriminatory performance for severe ARDS. Calibration (calibration curve): The fitted curve of observed versus predicted probabilities (green solid line) closely approximates the ideal line (blue dashed line) overall, with only slight deviation in the high-prediction-probability region. This indicates good overall model calibration, allowing for relatively accurate prediction of severe ARDS risk. Clinical utility (decision curve): When the threshold probability ranged from 0.01 to 0.8, the model’s net benefit (red curve) consistently exceeded that of the “no intervention” strategy (grey curve), with a particularly pronounced advantage in the 0.1–0.5 threshold probability interval. This suggests potential utility in identifying high-risk patients.3. Validation of the Nomogram for Intubation Risk in the Overall Cohort (**Figure 8C**). Discrimination (ROC curve): The model achieved an AUC of 0.971 (95% CI: 0.958–0.983), indicating excellent predictive accuracy for intubation requirement. Calibration (calibration curve): The fitted curve of observed versus predicted probabilities (green solid line) closely approximates the ideal line (blue dashed line), demonstrating minimal calibration error and strong agreement between model-predicted probabilities and actual intubation risk. Clinical utility (decision curve): When the threshold probability ranged from 0.02 to 0.7, the model (red curve) showed a significantly greater net benefit than the “no intervention” strategy (grey curve). Net benefit peaked within the 0.2–0.6 threshold probability range, suggesting potential clinical utility for intubation risk stratification. In summary, all three nomograms (predicting ARDS onset, severe ARDS, and intubation risk) demonstrated excellent discrimination, calibration, and clinical utility. AUC values exceeded 0.97 across all models, with strong goodness-of-fit in calibration curves. Decision curves validated their net benefit advantage within reasonable threshold ranges, suggesting these models serve as effective tools for ARDS risk stratification and management in clinical practice.

## 4 Discussion

COVID-19-associated acute respiratory distress syndrome (CARDS) carries a high mortality rate characterized by poor oxygenation accompanied by severe lung inflammation and damage (Cuerpo et al., 2025). Previous findings implicated the glycosaminoglycan (GAG) hyaluronan (HA) as a potential cause of fatalities in COVID-19 due to its ability to form a gel-like structure with high viscosity present in respiratory secretions in several inflammatory lung diseases (Li et al., 2022; Garantziotis et al., 2016; Bell et al., 2019; Hellman et al., 2020). HA has emerged as a component of the dysregulated immune response associated with COVID-19 (Albtoush and Petrey, 2022; Queisser et al., 2021; Barnes et al., 2023).

Our retrospective cohort analysis of 502 COVID-19 patients (361 in the non-ARDS group and 141 in the ARDS group) systematically evaluated the utility of hyaluronic acid (HA) in early ARDS diagnosis, severity stratification, and risk assessment of intubation, culminating in the development of a nomogram model. These findings suggest that HA may provide complementary value for ARDS-related risk stratification. Our results demonstrate a positive correlation between HA levels and disease severity: HA concentrations markedly increase with ARDS severity, exhibiting excellent diagnostic performance for ARDS (AUC = 0.904; sensitivity 81.6%, specificity 87.5% at a cut-off of 103 μg/L). HA also showed good discrimination for endotracheal intubation risk in the overall cohort: levels in the intubated group were significantly higher than in the non-intubated group (P < 0.001), with an AUC of 0.890 for predicting intubation requirement (sensitivity 76.6%, specificity 90.8% at a cut-off of 140.1μg/L; negative predictive value = 95.5%). Furthermore, HA demonstrated excellent discrimination in stratifying severe ARDS: its AUC for predicting severe ARDS was 0.953, numerically exceeding traditional markers such as CK and CRP, and showing a slightly higher AUC than SOFA and APACHE II scores (Basiri et al., 2025; Cylwik et al., 2024; Li et al., 2024a). Moreover, HA levels modestly improved multi-model prediction: integrating HA with clinical scores improved predictive performance—adding HA to the baseline model (age, lymphocyte count, CRP, CK, APACHE II, and SOFA) increased the AUC for severe ARDS prediction from 0.960 to 0.973 (ΔAUC = +0.013). Multivariate regression analysis confirmed HA as an independently associated factor for ARDS occurrence (OR = 1.04, P = 0.007), severe ARDS (OR = 1.00, P < 0.001), and intubation requirement (OR = 1.00, P = 0.011).

Similar to our findings, hyaluronic acid (HA) levels significantly increase during ARDS and remain elevated (Borrmann et al., 2023). SOFA and APACHE II scores are strongly correlated with ARDS severity. This association aligns with the systemic symptoms observed during ARDS and is valuable for assessing disease severity and prognosis. In our study, HA levels begin to rise during the early stages of ARDS, whereas SOFA and APACHE II scores remain low in patients with mild ARDS. Although the area under the curve (AUC) values for SOFA and APACHE II at ARDS diagnosis were 0.991 (95% CI: 0.983–0.999) and 0.992 (95% CI: 0.985–1.000), respectively, indicating near-perfect discriminatory capability. Hyaluronic acid showed good discrimination with an AUC of 0.904 (95% CI: 0.870–0.938). However, the SOFA and APACHE II scoring systems incorporate multiple factors and rely on patient symptom presentation. Despite their widespread clinical adoption, practical challenges remain in their assessment. Given that HA is a single factor independent of patient symptoms, it offers advantages in identifying occult ARDS patients and rapidly assessing ARDS severity. Therefore, the diagnostic value of HA for ARDS should not be overlooked. Accordingly, HA may be best interpreted as a complementary biomarker rather than a replacement for established clinical severity scores.

In our study, hyaluronic acid (HA) levels were significantly lower in patients with mild acute respiratory distress syndrome (ARDS) compared to those with severe ARDS, suggesting that HA levels reflect ARDS severity (Yu et al., 2024). Furthermore, our findings indicate that HA levels show a numerically slightly higher AUC than the SOFA and APACHE II scores in stratifying severe ARDS. Our findings also support an association between HA levels and endotracheal intubation risk (Li et al., 2024b). Considering the current clinical use of non-invasive ventilation and high-flow oxygen therapy, patients were classified according to whether invasive mechanical ventilation was required during hospitalization. The results indicate that HA levels significantly differentiate between these groups. These findings suggest that HA may contribute to respiratory risk stratification in clinical practice. This study has several limitations. First, it was a retrospective single-center study, which may limit the generalizability of the findings. Second, the moderate ARDS subgroup was relatively small, which may have reduced the precision of severity stratification. Third, plasma HA was measured only once within 24 hours of admission, and dynamic changes over time were not evaluated. Fourth, HA was analyzed as a continuous variable without categorized risk strata, which may limit the clinical interpretability of the estimated odds ratios. Finally, although internal validation was performed, external validation was not available. Future studies should further evaluate the temporal dynamics of HA, refine clinically meaningful HA risk strata, and validate these findings in larger multicenter cohorts.

## 5 Conclusion

This study demonstrates a positive correlation between HA levels and disease severity. HA concentrations markedly increase with the severity of ARDS, exhibiting excellent diagnostic performance for ARDS. Additionally, HA showed good discrimination for endotracheal intubation risk in the overall cohort. HA demonstrates excellent discrimination in stratifying severe ARDS, with a numerically slightly higher AUC than traditional markers such as CK and CRP, and with performance comparable to conventional SOFA and APACHE II scores. When combined with established clinical severity scores, HA modestly improved model performance and may serve as a complementary biomarker for early risk stratification in hospitalized COVID-19 patients. External validation is warranted before broader clinical application.

## Data Availability

The raw data supporting the conclusions of this article will be made available by the authors, without undue reservation.
