# Supplementary material for: A nomogram for predicting ARDS progression and need for invasive ventilation in COVID-19 patients using plasma hyaluronic acid and clinical scores
Source: Front Cell Infect Microbiol. 2026 Apr 16;16:1762512. doi: 10.3389/fcimb.2026.1762512 (PMC13128578; doi:10.3389/fcimb.2026.1762512)
Supplement: Supplementary file 1 [file Table1.docx]

**Hyaluronic Acid (HA) ELISA Detection Method**

**Step 1: Reaction System Construction**

**Addition of Samples and Antibodies:**

Add samples and pre-coated hyaluronic acid (HA)-specific antibodies to the wells of the ELISA plate (Human Hyaluronic Acid, ELK Biotechnology, ELK1234). The HA in the samples specifically binds to the antibodies coated on the plate.

Simultaneously, add biotin-labeled HA detection antibodies, which further bind to the HA already attached to the plate, forming a complex of “antibody-HA-biotin-labeled antibody.”

**Incubation:**

Cover the plate with a sealing film and incubate at 37℃ for 30 minutes to allow the reaction to proceed fully.

**Step 2: Washing**

**Removal of Unbound Substances:**

Carefully remove the sealing film, discard the liquid, and dry by shaking.

Add washing buffer to each well, let it stand for 30 seconds, then discard. Repeat this washing process 5 times and pat dry. This step removes unbound samples and antibodies to reduce background interference.

**Step 3: Signal Triggering and Measurement**

**Addition of Enzyme-Labelled Reagent:**

Add horseradish peroxidase (HRP)-labelled streptavidin (Streptavidin-HRP) to each well. This enzyme-labeled reagent specifically binds to the biotin-labeled antibody, forming a complete immune complex of “antibody-HA-biotin-labeled antibody-Streptavidin-HRP.”

**Color Development Reaction:**

Add color development reagents A and B to each well, gently mix, and incubate at 37℃ in the dark for 10-15 minutes. The color development reagent undergoes an oxidation-reduction reaction catalyzed by HRP, resulting in a color change.

**Termination of Reaction and Measurement:**

Add stop solution to each well to terminate the color development reaction. The stop solution is usually an acidic solution that halts the color development reaction.

Immediately measure the optical density (OD value) of each well at a wavelength of 450 nm using an ELISA reader (DNM-9602 analyser, Beijing Perlong New Technology Co., Ltd., China).

**Step 4: Result Calculation**

**Calibration Curve:**

Calculate the average OD values of the standards and sample replicates, and subtract the OD value of the blank well as the corrected value.

Plot the standard curve with the standard concentrations on the x-axis and the OD values on the y-axis, fitting a four-parameter logistic function on a double-logarithmic coordinate axis.

**Sample Concentration Calculation:**

Determine the concentration of the samples by locating their OD values on the standard curve. If the OD value of a sample exceeds the upper limit of the standard curve, the sample should be diluted appropriately and retested, with the dilution factor applied in the calculation.
